# Polysaccharide of *Taxus chinensis* var. *mairei* Cheng et L.K.Fu attenuates neurotoxicity and cognitive dysfunction in mice with Alzheimer’s disease

**DOI:** 10.1080/13880209.2020.1817102

**Published:** 2020-09-24

**Authors:** Senwei Zhang, Lulu Li, Jinting Hu, Ping Ma, Huimin Zhu

**Affiliations:** a School of Basic Medicine, Chengdu University of Traditional Chinese Medicine, Chengdu, China; b Zhejiang Provincial Hospital of TCM, Hangzhou, China; c Department of Microbiology and Immunology, Chengdu University of Traditional Chinese Medicine, Chengdu, China; d Department of Geriatrics, Taizhou Central Hospital, Taizhou, China

**Keywords:** Oxidative stress, apoptosis, spatial learning ability

## Abstract

**Context:**

Polysaccharide of *Taxus chinensis* var. *mairei* Cheng et L.K.Fu (Taxaceae) (PTM) functions in anti-apoptosis and antioxidation, but its function on Alzheimer’s disease (AD) remains unclear.

**Objective:**

To investigate the effect of PTM on AD.

**Materials and methods:**

C57BL/6J mice were randomly divided into three groups: control, d-galactose (d-gal), and d-gal + PTM. AD-like symptom was induced by d-gal for 6 weeks, followed with PTM (0.4 g/kg/d) for 14 days. PTM was added to BV2 cells stimulated with d-gal (1, 10, 20, 50, 100 and 500 μg/mL). Cell viability was evaluated by MTT assay. The expression of NRF2, SOD, cleaved caspase-3, Bax and Bcl-2 were detected with Western blot analysis. Cognitive function was evaluated by Morris water maze test.

**Results:**

Decreased cleaved caspase-3 (1.30 ± 0.09) and Bax/Bcl2 ratio (1.32 ± 0.11) were observed in BV2 cells induced by d-gal + PTM (50 μg/mL). Increased MDA and ROS and decreased SOD were observed in d-gal group. However, decreased MDA (175 ± 9 ng/mL) and ROS level (188 ± 38 ng/mL) were observed after treated with PTM group (*p* < 0.05). In addition, the expression of NRF2 decreased in d-gal group (0.75 ± 0.09) but increased after treated with PTM (*p* < 0.05). Furthermore, decreased Aβ1-42 was observed and the cognitive function was improved after PTM intervention (*p* < 0.05).

**Conclusions:**

This is the first report that PTM inhibited oxidative stress and apoptosis in AD. The result will further accelerate the applications of *Taxus chinensis* var. *mairei* and the treatment for AD.

## Introduction

Alzheimer’s disease (AD) is a degenerative disease of the central nervous system, characterized by progressive memory impairment and cognitive impairment accompanied by decreased daily living ability (Huang and Mucke 2012; Wilson et al. 2012). The incidence of AD increases sharply with age, accounting for about 60–70% of dementia cases (Fratiglioni et al. 2007). Generally, the prevalence of AD is 1% in population under 65, 16% in people aged 65–74, and 44% in elderly aged 75–84 (Ferri et al. 2005; Hebert et al. 2013). Thus, due to the aging population, AD has become one of the major global public health and social challenges of the future (Suh et al. 2016; Wimo et al. 2017). A variety of factors have been identified to be associated with AD, including heredity, neurotransmitter metabolism, neuronal apoptosis, free radicals, inflammation in the brain, immune dysfunction, endocrine, trace elements, poisoning, environment, poor cerebral circulation, and energy metabolism disorders (Cummings 2003). However, the aetiology and pathogenesis of AD remain unclear. Current drugs for AD, such as cholinesterase inhibitors and glutamate receptor blockers, could only improve the cognitive and behavioural abilities of AD patients to a certain extent instead of stopping and reversing the course of the disease (Citron 2002; Huang and Mucke 2012). Therefore, searching for drugs that can effectively delay the development of disease has become one of the key problems to be solved and overcome.

Polysaccharide is one of the natural macromolecular substances widely existing in higher plants, membrane of the animal cell and the cell walls of microorganisms, which plays an important role in maintaining life activities. Recently, polysaccharides, isolated from natural resources, have attracted great attention for their variety of pharmacological activities, such as antitumor, immunoregulation, antioxidation and anti-inflammatory effects (Li et al. 2012; Cho et al. 2015). Interestingly, polysaccharides of *Lycium barbarum* L. (Solanaceae) were reported to ameliorate the symptoms of AD mice and enhance neurogenesis in the hippocampus and subventricular zone, improving learning and memory abilities (Cheng et al. 2015). Previous studies demonstrated that the polysaccharide of *Taxus chinensis* var. *mairei* Cheng et L.K.Fu (Taxaceae) (PTM) fulfilled various biological functions, such as antitumor (Zheng et al. 2014; Wu et al. 2015), antioxidation, immune regulation and alleviation of myocardial ischemia-reperfusion injury (Zhu et al. 2011). Nevertheless, whether PTM could attenuate the symptoms of AD remains largely undetermined. In the present study, we established the AD model to investigate the protective role of PTM, which might provide a new treatment for AD.

## Material and methods

### PTM ingredients and sources

PTM were extracted from the branches and leaves of *Taxus chinensis* var. *mairei* with ion-exchange and gel-permeation chromatography method according to the published reference (Wu et al. 2009). PTM possesses a 1,3,6-linked β-d-Manp main chain, which were substituted by mannose, arabinose, galactose, rhamnose, xylose,2,4-di-*O*-methyl-mannose, galacturonic acid, and glucuronic acid residues. These contained non-reducing end-units of mannose, arabinose, xylose, 2,4-di-*O*-methyl-mannose, and glucuronic acid (Wu et al. 2009).

### Animal experiments

C57BL/6 mice aged 8 weeks were purchased from the animal model centre of Nanjing University (Nanjing, China). All animal experiments were carried out in accordance with the National Institutes of Health Guide for the Care and Use of Laboratory Animals and were approved by the animal care and use committee of School of Clinical Medicine, Traditional Chinese Medicine of Chengdu University (TCMCULL2019068593). Mice were randomly divided into 3 groups: control group, d-gal group and d-gal + PTM group. d-Gal was used to induce AD-like symptom in mice via intraperitoneal injection at a dose of 150 mg/kg/d for 6 weeks, PTM was given to the mice via intragastric administration at a dose of 0.4 g/kg/d for 14 days after d-gal injection.

#### Cell culture and treatment

Cell line BV2 was cultured in high glucose Dulbecco’s modified Eagle’s medium (DMEM) supplemented with 10% foetal bovine serum (FBS) and 5% horse serum, 1% penicillin/streptomycin at 37 °C in incubator with 5% CO_2_. After 24 h. cells were pre-treated with PTM (Hangzhou, China) for 48 h, followed with d-gal (1 M) (Sigma-Aldrich, St. Louis, MO). treatment for additional 48 h. Transfection of siRNA (75 nM) was conducted using Lipofectamine 3000 (Invitrogen, MA) according to the manufacturer’s instruction.

#### Cell viability assay

Cell viability was evaluated by the MTT reduction assay. Briefly, BV2 cells were cultured in 96-well plates and exposed to d-gal. Subsequently, PTM was added to BV2 cells in different concentrations (1, 10, 20, 50, 100 and 500 μg/mL) for 48 h. MTT (40 μL) was added and incubated for 4 h, and the absorbance of cells was measured using a microplate reader at 490 nm (Thermo Fisher, Waltham, Ma). Cell viability was expressed as percentage of the absorbance of treated cells relative to control cells.

#### Western blot analysis

Protein was extracted from cells or brain tissues and lysed in RIPA buffer (Sigma, St. Louis, MO). Equal quantity of protein was exposed to 12% SDS-PAGE gels and transferred to PVDF membranes (Roche Diagnostics, Mannheim, Germany). The primary antibodies were as followed: Bax, B-cell lymphoma/leukemia 2 (Bcl-2), Cleaved caspase-3, superoxide diamutase (SOD) and nuclear factor E2-related factor 2 (NRF2). GAPDH was used as the loading control.

## Enzyme-linked immunosorbent assay (ELISA)

To determine the effect of PTM on AD-related toxic protein (Aβ1-42) and oxidative stress markers (MDA and ROS), BV2 cells were pre-treated with PTM for 2 h prior to incubate with d-gal for 24 h. Homogenate of BV2 cells were lysed and centrifuged at 15,000 *g* for 15 min to remove the debris, then concentrated with an ultrafiltration tube (Millipore, Billerica, CA). MDA and SOD content in the supernatant fluid from BV2 cells and brain tissue were determined using ELISA kits for MDA according to the manufacturer’s instructions. The MDA content was measured based on absorbance values of samples were measured at 450 nm in an ultraviolet spectrophotometer (UVmini-1240). The Aβ1-42 ELISA Kit was used to determine expression of Aβ1-42 according to the manufacture’s protocol (Zhou et al. 2018).

### Hematoxylin–eosin (HE) and immunohistochemical staining

To observe the histopathological changes of brain tissues in AD mice, brain tissues were isolated after mice sacrificed. Subsequently, the above tissues were embedded with paraffin, cut into sections and treated with immunohistochemical staining and HE staining. Finally, the sections were captured using microscope (NIKON, Tokyo, Japan).

### Morris water maze test

Morris water maze test (MWM) was applied to evaluate the role of PTM on spatial learning ability of mice with AD which induced by d-gal. Progress of MWM test was performed in accordance with the published references (Vorhees and Williams 2006). The escape latencies and platform-crossing times were recorded and analyzed.

### Statistical analysis

All data were analyzed and presented as mean ± SD by using Graph pad prism 7.0 (GraphPad Software, La Jolla, CA). Comparisons among groups were assessed using one-way ANOVA followed by Bonferroni’s *post hoc* test. Comparisons within groups were assessed using multivariate tests and *p* < 0.05 was considered statistically significant.

## Results

### Protective effects of PTM on viability of BV2 cells stimulated with d-gal

**Figure 1.**
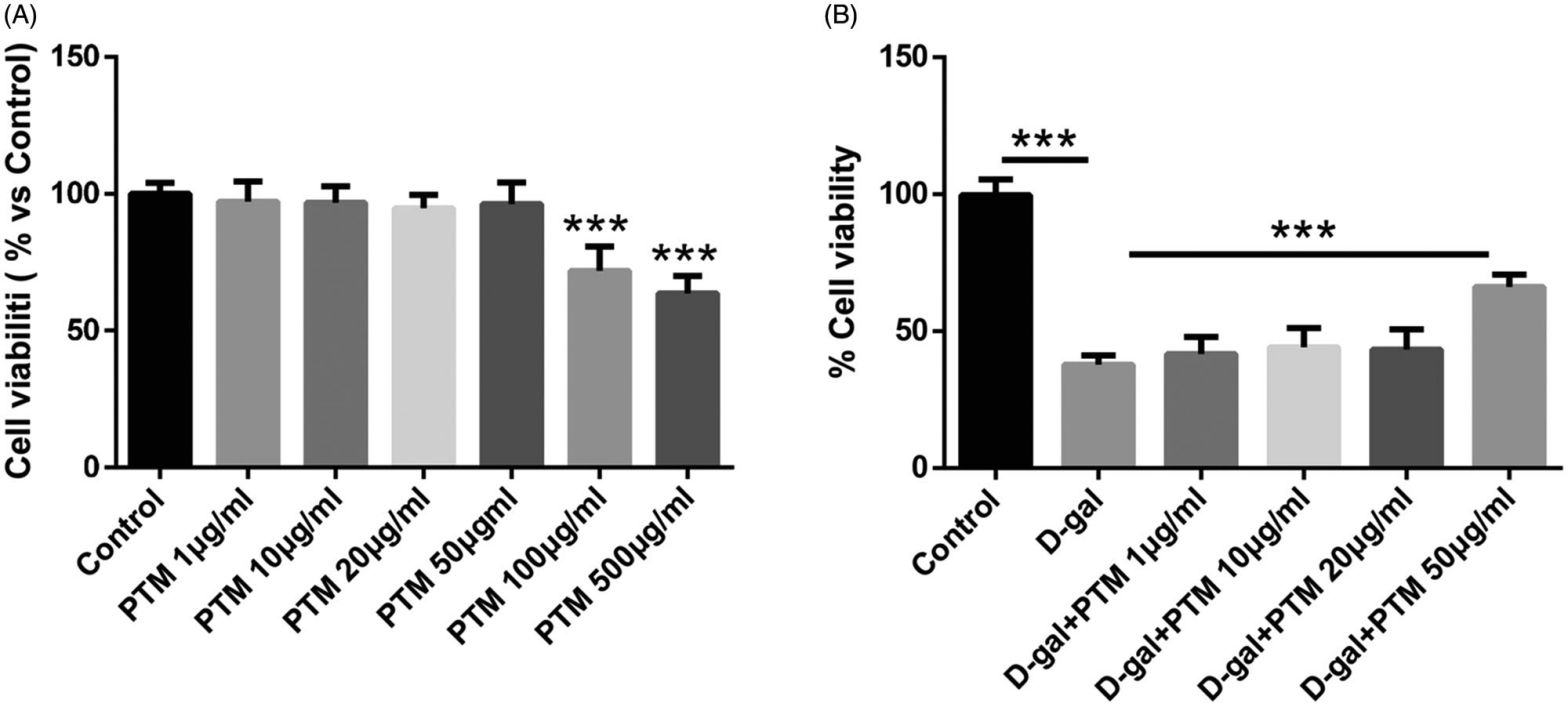
Polysaccharide of *Taxus chinensis* var. *mairei* (PTM) pre-treatment protected BV2 cells against d-gal-induced cell death.

To screen the suitable concentration of PTM in BV2 cells, MTT assay was carried out to detect the effect of different concentrations of PTM on cell stability induced by d-gal. As shown in Figure 1(A), PTM exerted no significant cytotoxic effect at concentrations up to 50 μg/mL after 24 h treatment. Concentrations of PTM at 100 and 200 μg/mL resulted in a marked decrease in cell stability (*p* < 0.05). In addition, we pre-treated cells with different concentrations of PTM (1, 10, 20 and 50 μg/mL) and then treated cells with 10 mM concentration of d-gal for 24 h. MTT assay confirmed that PTM at 50 μg/mL concentration could significantly improve the stability of cells decreased by d-gal (Figure 1(B)) (*p* < 0.001). Therefore, we selected 50 μg/mL as the concentration of PTM in this study.

### PTM alleviated apoptosis and oxidative stress induced by D-gal in BV2 cells

**Figure 2.**
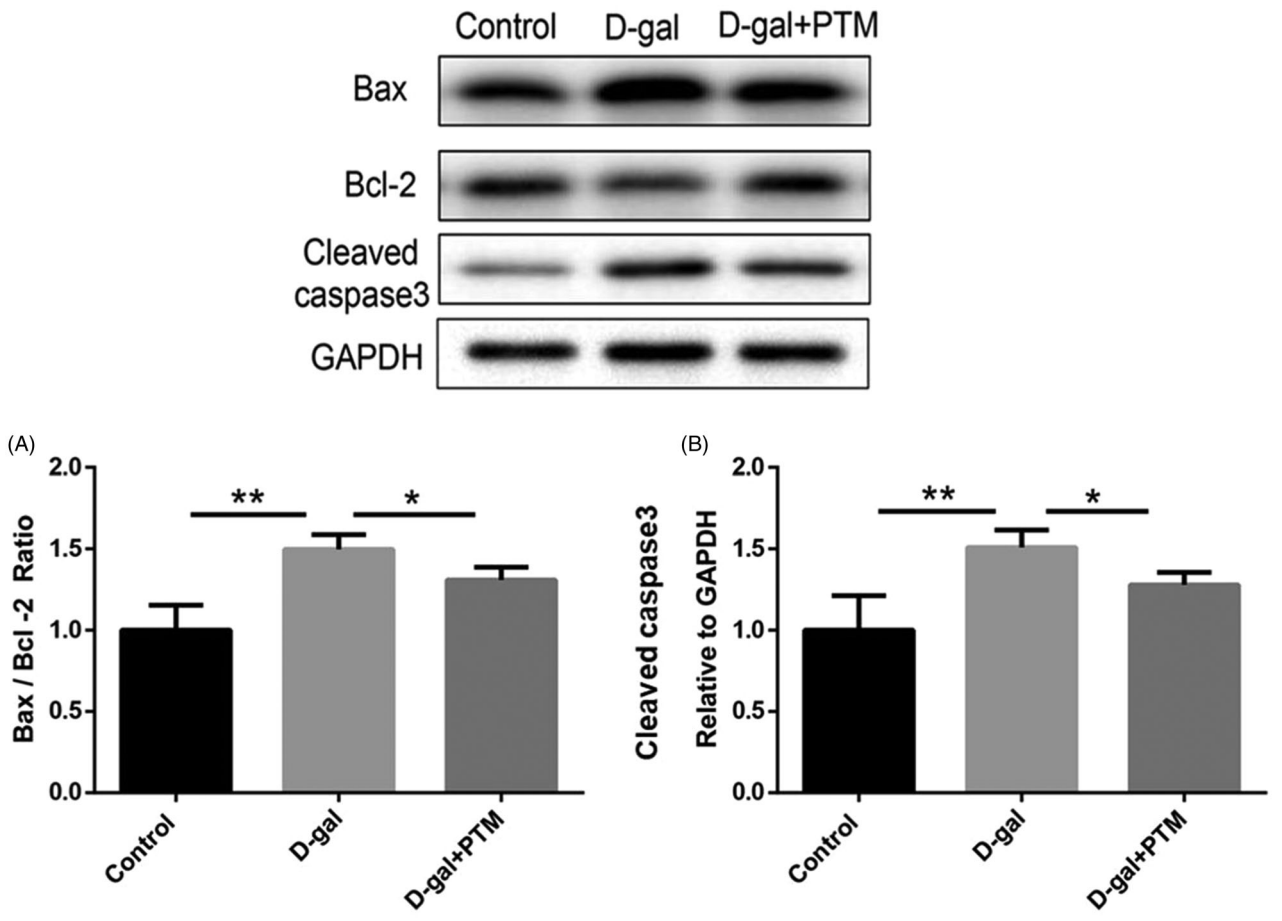
Polysaccharide of *Taxus chinensis* var. *mairei* (PTM) decreased attenuated the d-gal induced apoptosis in BV2 cells.

**Figure 3.**
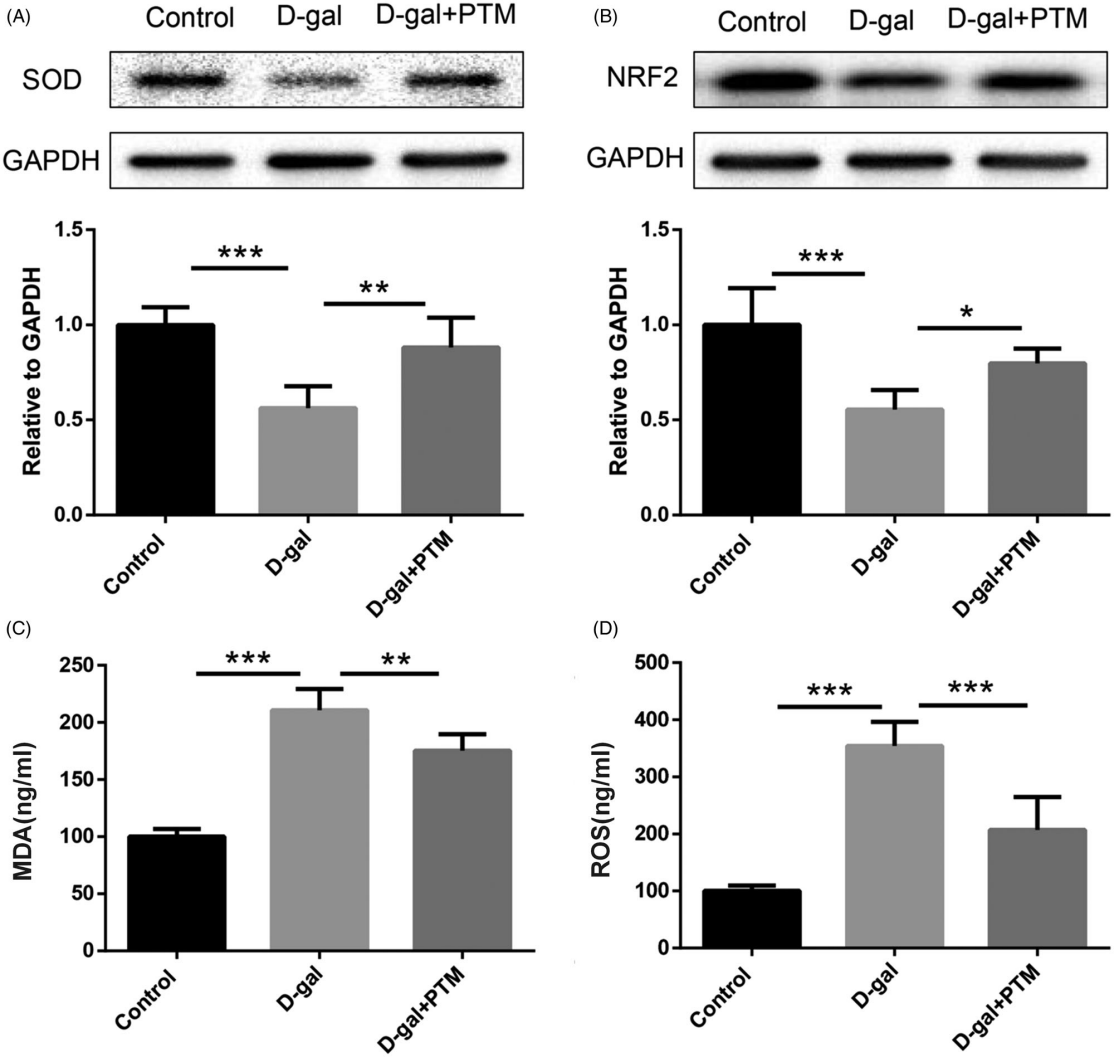
Effects of polysaccharide of *Taxus chinensis* var. *mairei* (PTM) on oxidative stress in BV2 cells treated with d-gal.

To determine the effect of PTM on BV cells induced by d-gal, the apoptosis related markers (Bax, Bcl2 and cleaved caspase-3) and the oxidative stress markers (MDA, SOD, and ROS) were measured. Compared with control group, high expression of Bax/Bcl-2 ratio (Figure 2(A)) and cleaved caspase-3 (Figure 2(B)) in Western blot analysis were observed in BV2 cell with d-gal. Interestingly, PTM significantly inhibited the risen level of apoptosis-related proteins, including Bax/Bcl-2 ratio and cleaved caspase-3 in BV2 cells which irrigated by d-gal (*p* < 0.05) (Figure 2(B)). Similarly, the expression of SOD was observed to be significantly inhibited by PTM in BV2 cells when exposed to d-gal (*p* < 0.001) (Figure 3(A)). In addition, compared with control group, the level of MDA and ROS production were elevated in d-gal induced BV2 cells, while the up-regulation of MDA level and ROS production were significantly attenuated by PTM (*p* < 0.05) (Figure 3(C and D)). Moreover, Western blotting analysis demonstrated that NRF2, an important regulator of oxidative stress, was down-regulated in the d-gal-treated group when compared with control group, but the down-regulation of NRF2 expression was reversed by PTM (*p* < 0.05) (Figure 3(D)).

### Inhibitory effect of PTM on d-gal-induced BV2 cells was blocked after NRF2 silenced

**Figure 4.**
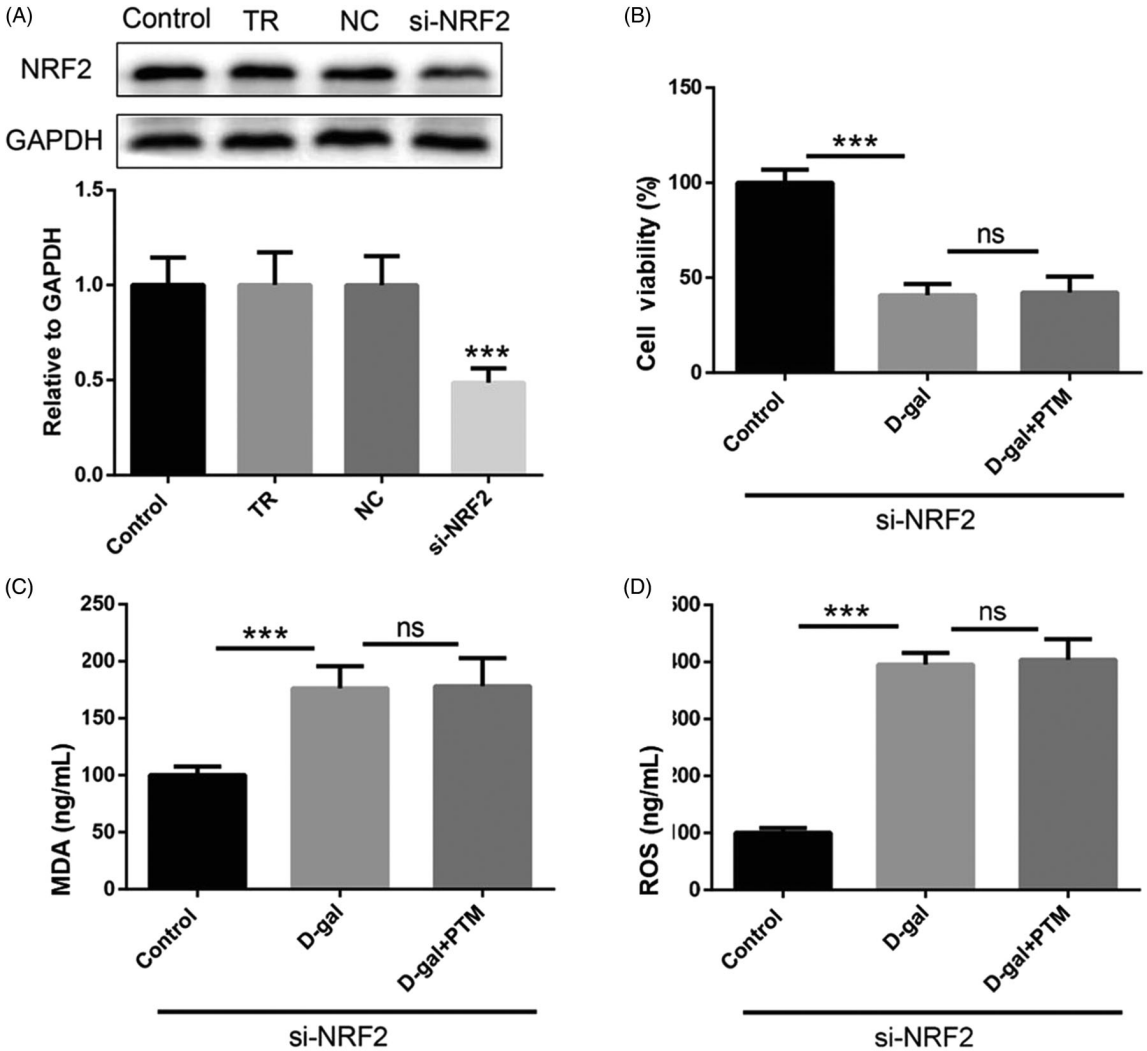
Si-NRF2 abrogated effect of polysaccharide of *Taxus chinensis* var. *mairei* (PTM) on d-gal-treated cells.

**Figure 5.**
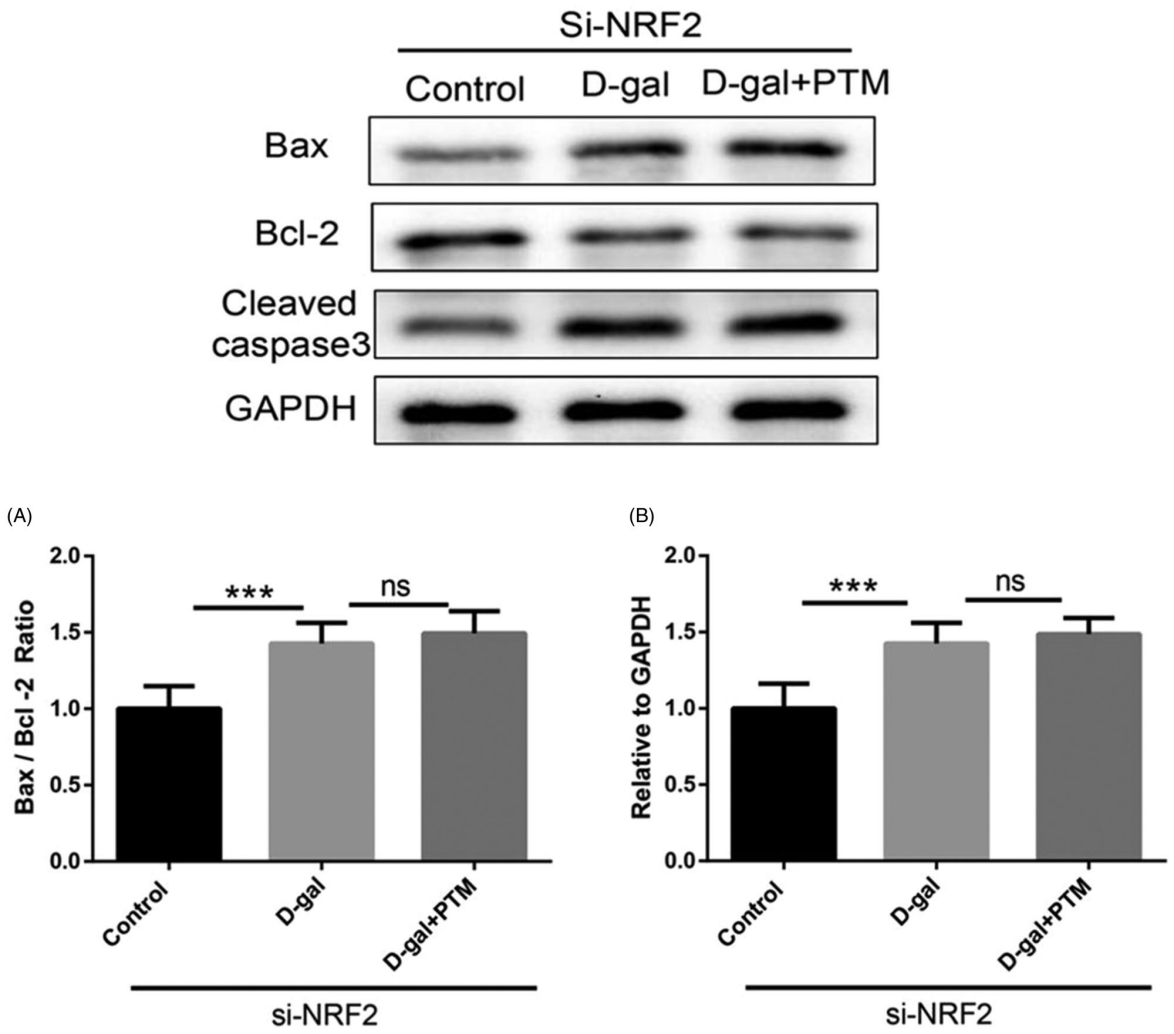
Si-NRF2 diminished benefits of polysaccharide of *Taxus chinensis* var. *mairei* (PTM) on d-gal-treated cells.

To further explore the mechanism of the reducing oxidative stress in AD mice by PTM, we investigated the role of NRF2 in the inhibitory effect of PTM on d-gal-induced BV2 cells, we used NRF2 siRNA to down-regulate the NRF2 expression (Figure 4(A)). MTT assay results showed that the protective effect of PTM on d-gal-induced decrease in cell stability was blocked after NRF2 silenced (*p* < 0.001) (Figure 4(B)). In addition, the suppressed role of PTM on d-gal-induced oxidative stress in BV2 cells was partially reversed after NRF2 silenced (*p* < 0.05) (Figure 4(C)). Consistent with the above results, the level apoptosis related proteins (Bax, Bcl2 and cleaved caspase-3) increased in d-gal-treated BV2 cells, which were not significantly attenuated by PTM (Figure 5(A,B)).

### PTM improves histopathological changes and D-gal induced injured brain in mice

**Figure 6.**
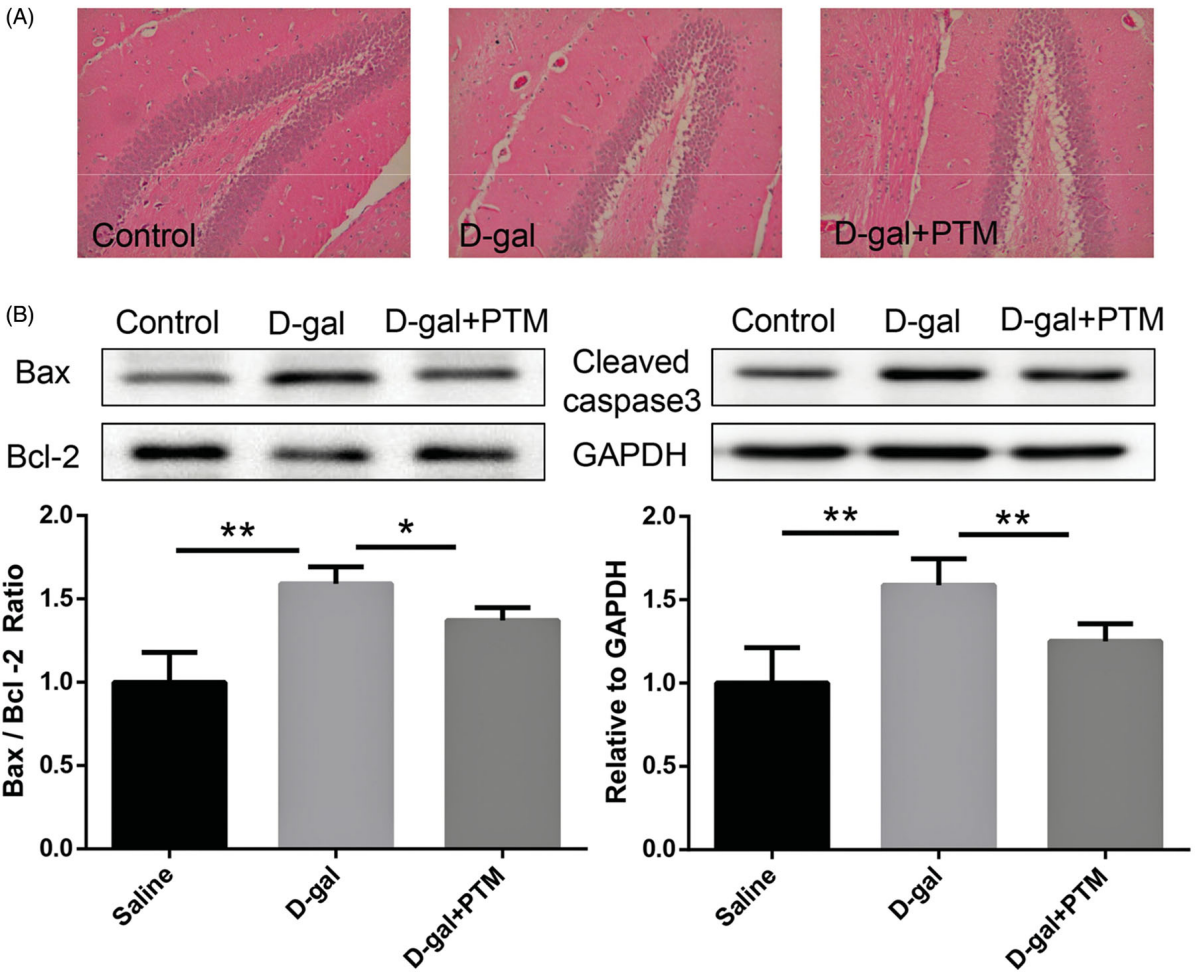
Polysaccharide of *Taxus chinensis* var. *mairei* (PTM) improved histopathological changes and d-gal induced apoptosis in mice brain.

To evaluate the effect of PTM on d-gal induced brain injury in mice, HE staining and Western blot analysis were performed in this study. HE staining results showed that the hippocampal pyramidal cells in the d-gal-treated group were sparsely arranged, disordered and the number of cells was decreased compared with the control group, whereas the brain injury in AD-like mice induced with d-gal was alleviated by PTM (Figure 6(A)). Moreover, the expression of caspase-3 and Bax/Bcl-2 ratio increased in AD-like mice (*p* < 0.01). However, PTM significantly inhibited the risen level of apoptosis-related proteins, including cleaved caspase-3 and Bax/Bcl-2 ratio, which were irrigated by d-gal (*p* < 0.05) (Figure 6(B)).

### PTM increased NRF2 expression and decreased oxidative stress reaction

**Figure 7.**
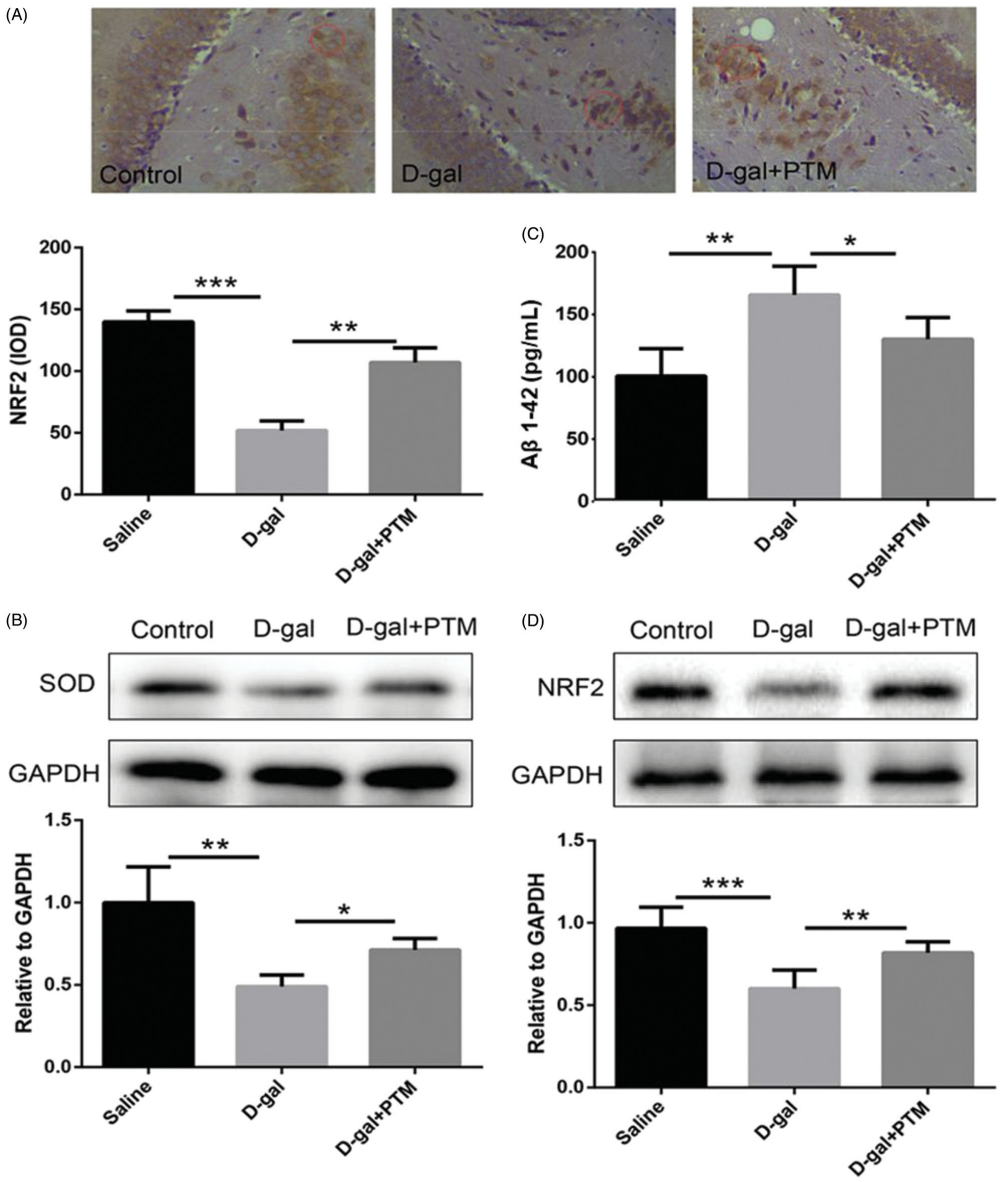
Polysaccharide of *Taxus chinensis* var. *mairei* (PTM) increased NRF2 expression and decreased oxidative stress.

In this study, we further confirmed the role of PTM on NRF2 protein expression and oxidative stress induced by d-gal in mice brain. Immunohistochemical staining showed that the PTM significantly increased the expression of NRF2 inhibited by d-gal (*p* < 0.01) (Figure 7(A)). In addition, PTM suppressed the toxic protein Aβ1-42 expression and apoptosis induced by d-gal in AD mice. Increased Aβ1-42 level (161 ± 11.2 pg/mL) was observed in AD-like mice induced by d-gal when compared with control group (saline), but decreased Aβ1-42 level was observed after PTM intervention (*p* < 0.05) (Figure 7(C)). In addition, the western blotting analysis confirmed that the expression of SOD (*p* < 0.01) (Figure 7(B)) and NRF2 were inhibited in AD-like mice but rescued by PTM intervention (*p* < 0.05) (Figure 7(D)).

### PTM improved the spatial learning ability in AD-like mice

**Figure 8.**
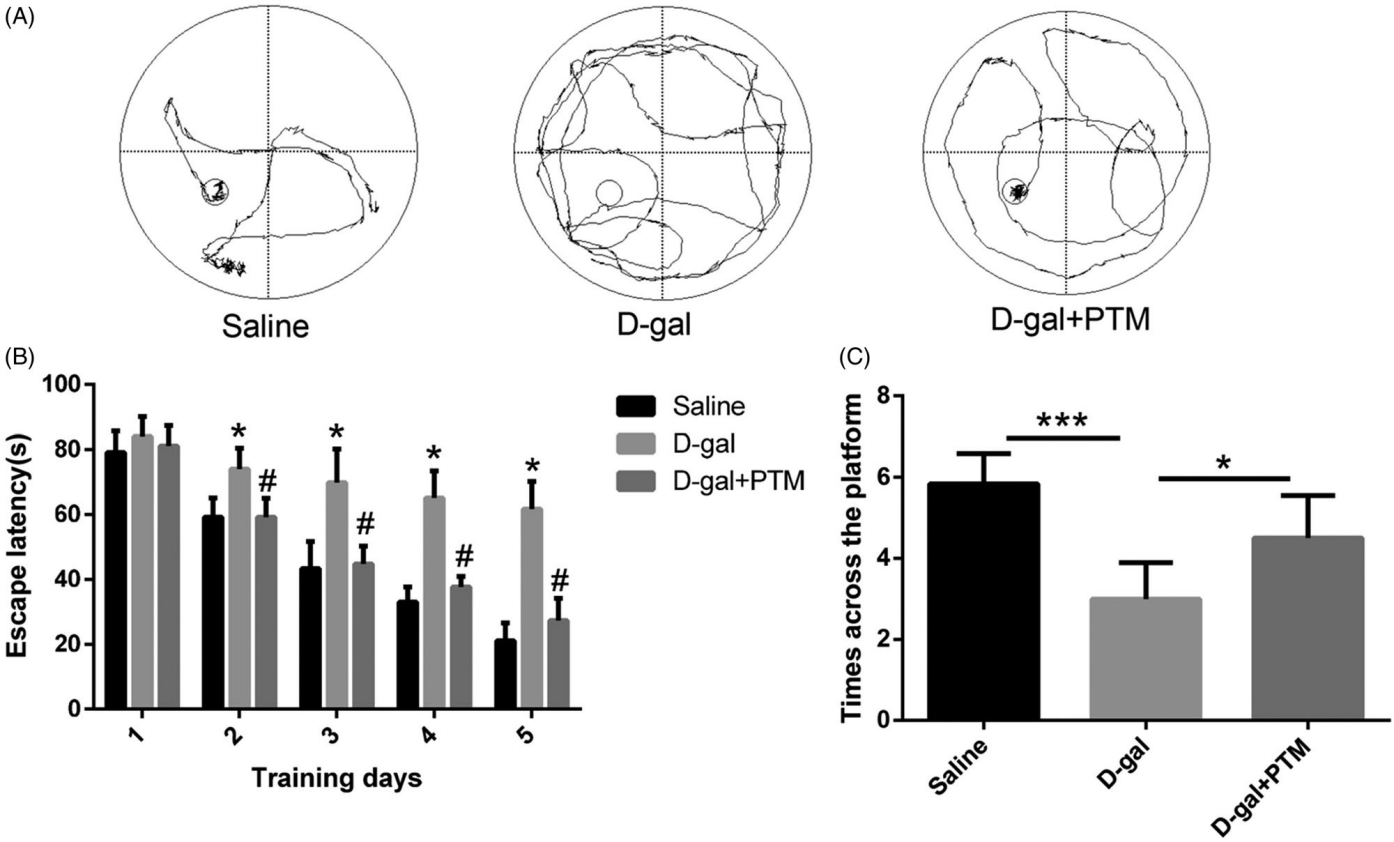
Polysaccharide of *Taxus chinensis* var. *mairei* (PTM) attenuated d-gal induced recognition impairments in mice.

As shown in Figure 8, the navigation paths data revealed that spatial learning ability of AD-like mice induced by d-gal was markedly decreased compared with the control groups (*p* < 0.05). However, administration of PTM in AD-like mice significantly ameliorated spatial learning capability in d-gal-treated mice (*p* < 0.05) (Figure 8(A)). The longer of scape latencies and fewer number of platform crosses in d-gal-treated mice were obviously improved after PTM administration (*p* < 0.05) (Figure 8(B,C)). These results demonstrated that PTM could improve the cognitive function in d-gal-treated mice.

## Discussion

In the present study, to the best of our knowledge, we demonstrate for the first time that PTM can improve the spatial learning ability and cognitive function in d-gal-treated mice. The underlying mechanism may be related to suppressing Aβ expression and apoptosis and decreasing oxidative stress reaction via increasing NRF2 expression.

AD is one of the most common types of chronic neurodegenerative diseases. It manifests as memory deterioration and cognitive deficits, which eventually leads to physical dysfunction and death (Kumar et al. 2017). At present, the incidence of AD is increasing year by year all over the world. By 2050, the number of AD patients will reach 131 million (Chibnik et al. 2017; Hsiao et al. 2018). In fact, the prevention and treatment of AD have attracted great attention all over the world. However, the efficacy of current drugs for AD is limited and the specific pathogenesis of AD remains unknown. Interestingly, Aβ, oxidative stress and cell apoptosis have proven to participate in the pathological process of AD (Hung et al. 2018). Hence, to find new, efficient and low toxicity drugs act on these targets for the treatment of AD is vital important.

Polysaccharides isolated from natural resources have proven for their antioxidation and anti-inflammatory effects (Li et al. 2012; Cho et al. 2015). Previous studies reported that polysaccharides played an important role in the pathogenesis of AD (Cheng et al. 2015). For example, polysaccharide of *Lycium barbarum* could protect neurons from Aβ-induced apoptosis (Yu et al. 2007). In addition, 7-day oral administration of polysaccharide of *Lycium barbarum* effectively improved neurological deficits and protected the brain from BBB disruption (Yang et al. 2012). Consistent with our research, PTM administration alleviated the burden of oxidative stress injury and further improved the cognitive function and learning ability in AD.

Intertwining of neurofibrils, loss of neurons and senile plaque formation are important pathological features in the brain of AD patients (Chandra 2017). Aβ is considered to be the most important component of senile plaque (Chandra 2017). Excessive Aβ deposits outside the cortex and hippocampal neurons, forming plaque tangles that lead to abnormal synaptic function, loss of nerve cells and inflammation, eventually leading to neurological structural and functional disorders and the formation of dementia (Nasica-Labouze et al. 2015). Besides, abnormal Aβ is able to induce apoptosis and neurotoxicity (Lee et al. 2013; Porcellotti et al. 2015). Interestingly, Aβ may affect cell apoptosis by regulating the activity of caspase-3, which plays an irreplaceable role in the process of apoptosis (Chang et al. 2016). Our results indicated that PTM treatment could reduce Aβ expression and apoptosis-related protein cleaved caspase-3 and Bax/Bcl-2 ratio in AD-like mice.

In recent years, the role of oxidative stress in the pathogenesis of AD has gradually attracted more and more attention (Persson et al. 2014). Oxidative stress is one of the important factors in the progression of AD. It can be used as an important marker in the early stage of AD, even earlier than the deposition of Aβ (Persson et al. 2014). In fact, oxidative stress interacts strictly with the production of Aβ. On the one hand, oxidative stress increases the production and deposition of Aβ which in turn promotes the formation of senile plaques (Cheignon et al. 2018). On the other hand, Aβ exerts crucial function on the production of ROS, thus, speeding up the progress of AD (Kim et al. 2016). To determine whether PTM can alleviate d-gal induced oxidative stress, the expression levels of SOD, ROS and MDA were determined. Our results showed that the decrease expression levels of SOD protein, an antioxidant, was alleviated by PTM in AD mice. However, the increased level of MDA, a product of lipid peroxidation, and the increased level of ROS were significantly attenuated by PTM.

Nrf2, as one of the most important transcription factors in central nervous system, plays an important role in cell defense against oxidative stress (Liu et al. 2019). Once Nrf2 is stimulated by chemicals, more Nrf2 is produced and accumulated. Nrf2 then enters the nucleus to induce ROS degradation and protect cells from ROS damage (Yamamoto et al. 2008; Sasaki et al. 2013). In fact, Nrf2 has been proven for their various cytoprotective effects on anti-inflammatory, antitumor, antiatherosclerosis, anti-ischemia–reperfusion injury and anti-nerve injury (Liu et al. 2019). In present study, except for abnormally elevated oxidative stress, NRF2 was also down-regulated in the d-gal-treated group, however, PTM reversed such dramatic changes.

To further investigate and verify NRF2 in PTM protecting d-gal-treated BV2 cells, siRNA to down-regulate the NRF2 expression was then used in experiment. As shown in our study, si-NRF2 blocked the protective effect of PTM on d-gal-induced decrease in cell stability and suppressed the valuable role of PTM on d-gal-induced oxidative stress in BV2 cells. These results indicated that PTM inhibited Aβ deposition, apoptosis and oxidative stress via NRF2.

In order to further confirm the above capability, we evaluated the effects of PTM on cognitive function, Aβ deposition, apoptosis, oxidative stress and NRF2 expression in d-gal-induced AD mice. The Morris water maze is a widely used model for studying learning and cognitive function in mice, which assesses spatial learning and cognitive specifically (Barone et al. 2000; Crawley 2008). The results demonstrated that administration of PTM significantly ameliorated spatial learning capability in d-gal-treated mice. The hippocampus of AD patients is the first lesion area in the course of disease (Berlit et al. 2015). After injury, patients may experience the loss of memory and sense of direction (Berlit et al. 2015). It has been found that hippocampus-impaired monkeys suffered from severe impairments in memory of recently learned tasks (Zola-Morgan and Squire 1990). In the present study, the sparsely arranged, disordered and the decreasing number of hippocampal pyramidal cells induced by d-gal were significantly inhibited by PTM. Furthermore, consistent with the previous study, the data demonstrated that PTM inhibited Aβ deposition, impeded apoptosis, attenuated oxidative stress and reversed the NRF2 expression in d-gal-treated mice.

## Conclusions

This study demonstrated that PTM could indeed inhibit Aβ deposition, apoptosis and oxidative stress both in AD, and restore the impaired learning and cognitive function via regulating NRF2 expression. The result will further accelerate the applications of *Taxus chinensis* var. *mairei* and provide a novel idea for the treatment of AD.
